# The Gambling Consumption Mediation Model (GCMM): A Multiple Mediation Approach to Estimate the Association of Particular Game Types with Problem Gambling

**DOI:** 10.1007/s10899-020-09928-3

**Published:** 2020-01-21

**Authors:** Tim Brosowski, Daniel Thor Olason, Tobias Turowski, Tobias Hayer

**Affiliations:** 1 University of Bremen, Bremen, Germany; 2 University of Iceland, Reykjavík, Iceland

**Keywords:** Problem gambling, Intensity, Mediation, Breadth, Depth

## Abstract

Bivariate associations of problem gambling with participation in particular game types often decrease when adjusting for demographics or consumption behavior (e.g., number of game types played). A summary of 14 peer-reviewed studies showed inconsistencies as well as conceptual and methodological challenges. The aim of this study was to expand previous research by a combination of (1) sophisticated feature-engineering, which disaggregates gambling intensity into facets *within* and *beyond* a game type of interest, and (2) the application of mediation models. Data comprised last year gamblers of three merged cross sectional Icelandic gambling surveys of 2007, 2011, and 2017 (N = 4422). For each of 15 game types (12-month time frame), a parallel multiple mediation model was applied to disaggregate bivariate associations of last year game type participation and problem gambling (Problem Gambling Severity Index) by six mediating mechanisms: (1) demographic problem gambling propensity, (2) number of game types played, (3) gambling frequency within the type, (4) maximum gambling frequency across all types beyond, (5) usual spending within the type, (6) maximum usual spending across all types beyond. Games showed two distinct profiles via which mediator they mostly impacted problem gambling: Electronic gaming machines offline, scratch cards offline, live betting online, and poker offline as well as online impacted problem gambling mostly via gambling *frequency within*, whereas all other types mostly impacted via *the number of game types played*. The applied mediation models answer the question *by which mechanism* game types impact problem gambling in a more exhaustive way than previous research.

## Introduction

### A Quite Old Discussion About Problem Gambling, Game Type, and Gambling Behavior

In general, it is a well known fact that problem gamblers tend to invest more time and money into gambling and usually participate in a larger number of games than non-problem gamblers (National Research Council 1999; Welte et al. 2002). However, there is also an old but still ongoing debate about the potential risks posed to individuals by their participation in particular types of gambling (Shaffer and Martin 2011). Earlier research findings suggest that continuous games with an element of skill or perceived skill are more strongly associated with problem gambling than other types of games (Dowling et al. 2005; Griffiths 1999; Productivity Commission 1999). Moreover, longitudinal data from Canada provided evidence that participating in casino games or playing electronic gaming machines (EGMs) at least once a month is a valid predictor of progression from low-risk to high-risk gambling involvement (Currie et al. 2011). Similarly, the analysis of cross-sectional data from Germany (Brosowski et al. 2015) showed that even infrequent participation in EGMs (i.e., more than 2 days in the last year) reliably increased the risk of a current gambling disorder by a factor of 39. This result remained even after controlling for five demographic risk factors (age, gender, education, unemployment, migration background). Finally, data from diagnostic interviews in Germany revealed that gambling on EGMs was associated with suicidal events in pathological gamblers independently of comorbid disorders (Bischof et al. 2016).

### Common Explanations for Associations of Problem Gambling and Game Types

One way to explain the increased risks of problem gambling among patrons of specific game types is related to *structural or contextual characteristics* of the game types like availability or event frequency (Griffiths 1993; Meyer et al. 2011). Another explanation may be the *selective attractiveness* of particular game types for vulnerable individuals, for instance socio-economically disadvantaged populations (Rintoul et al. 2013; Wardle et al. 2014). However, rather recently, several authors have proposed an alternative way of explanation, using regression models with several predictors. By using this *regression*-*modeling approach,* researchers revealed strong bivariate associations between specific game types and problem gambling. Table 1 summarizes information of 14 peer-reviewed publications of this regression-based line of research from 2009 to 2018.

**Table 1:** Current peer-reviewed research on regression-based model estimates of covariates and game type specific risks between 2009 and 2018

References	Sample	N	Pivotal method	Predictors of problem gambling	Main results
#1 Welte et al. (2009)	Random telephone survey in the USA among adolescents (14–21 years old)	1535 last year gamblers	Simultaneous negative binomial regression of all game types	Participation in 15 game types (both tested: dichotomous or ordinal level), covariates (gender, age, socio-economic status), and interaction terms of forms with the three covariates	On a dichotomous participation level, the most important predictors were: card games, casino gambling, other games, and games of skill; on an ordinal participation level the most important predictors were casino gambling, office pools, and charity, other games, lottery, card games, and games of skill; significant interaction effects between gender and some game types
#2 LaPlante et al. (2011)	Secondary data analysis of the 2007 British Gambling Prevalence Survey; at least 16 years old	8968 last year gamblers	A series of logistic regressions for each game type	Participation in 15 game types (dichotomous level) as single predictors and also adjusted for the total number of game types a person played	Adjusting for the number of game types involved in strongly reduced the impact of each game type on problem gambling; the only significant predictor left after adjustment was participation in virtual gaming machines; private betting and betting on horses even became significant protective factors
#3 Afifi et al. (2010)	Secondary data analysis of the Canadian Community Health Survey 2002; only women, at least 15 years old	10,056 women	A series of logistic regressions for each game type	Participation in 15 game types (ordinal level) as single predictors and also adjusted for several covariates (age, income, education, marital status, life stress, social support, and negative coping)	The general trend noted in the results was that the probability of problem gambling increased with greater frequency of gambling in any game type; after adjusting for covariates, regular gambling involvement in video lottery terminals inside or outside casinos, other casino games, bingo and instant win tickets posed highest risks for problem gambling
#4 Brosowski et al. (2012)	Active customers of an online gambling provider; predictors extracted from actual gambling behavior across 7 months	27,653	A series of logistic regressions for each game type	Participation in 6 game types (dichotomous level) adjusted for the number of game types played	Adjusting for the number of game types only left live action betting and poker as significant risk factors for simultaneously transgressing two established offline-thresholds of probable at-risk gambling; each additional game type increased the transgression risks
#5 Haß et al. (2012)	Secondary data analysis of three German gambling surveys 2007, 2009, 2011; at least 16 years old	15,165 last year gamblers	A series of bivariate and simultaneous logistic regressions for all game types	Participation in 14 game types (dichotomous level) adjusted for (1) participation in all other game types, (2) total number of game types played, (3) covariates (age, gender, education, ethnicity, employment status)	After adjusting for covariates, number of game types involved in, and participation in all other game types simultaneously, lotto became a significant protective factor; highest risks were still posed by electronic gambling machines, Internet casino games, keno, casino table games (not slots), and other sport betting (private provider)
#6 LaPlante et al. (2013)	Survey of casino patrons at two Las Vegas resort casinos	676 last year gamblers	A series of logistic regressions for each game type	Participation in 10 game types (dichotomous level) adjusted for (1) the frequency of gambling across all game types during the last year, (2) the total number of game types played during the current visit), and (3) both	Frequency of play during the past year, rather than game played or number of games played during the current visit, consistently predicted a history of gambling problems
#7 LaPlante et al. (2014)	Active customers of an online gambling provider who responded to an online survey to assess problematic gambling behavior; predictors extracted from actual gambling behavior	1440	A series of logistic regressions for each game type	Participation in 16 game types (dichotomous level) as single predictors and also adjusted for (1) the total number of game types played and (2) the number of days a person placed a bet, (3) and both	Adjusting for the number of game types only left live action betting as a significant risk factor; after adjusting for the number of active gambling days the strongest predictors were phone-based casino software, simulated sports games, backgammon, downloadable casino software, games played in between bets, browser-based casino software, fortune games, and live action betting; after adjusting for both indicators of involvement poker even became a significant protective factor; both indicators were slightly correlated and the risk of problem gambling increased almost linearly with ascending levels of involvement
#8 Afifi et al. (2014)	Secondary data analysis of the Canadian Community Health Survey 2002; at least 15 years old	18,913 last year gamblers	A series of bivariate and simultaneous logistic regressions for all game types	Participation in 13 game types (ordinal level; partially applied dichotomously) (1) adjusted for participation in other types, (2) adjusted for other types and the total number of game types played, and (3) interactions of involvement with age and gender	After adjustment for participation in other game types and the number of game types, regular gambling increased the risks of almost all game types; irregular gambling in some game types posed a protective effect, for instance in instant win or lottery tickets; regular involvement in electronic gaming machines inside casinos, other casino games, and electronic gaming machines outside casinos posed the largest risks for problem gambling; the number of game types involved in did not interact significantly with age or gender
#9 Scalese et al. (2016)	Secondary data analysis of an Italian population survey from 2010–2011; 15–64 years old	5292 individuals who completed the Problem Gambling Severity Index	Ordinal logistic regressions in two separated samples: one-game-users and multi-game users	Participation in 5 game types (dichotomous level) adjusted for age, gender, employment status, and educational level	Being involved in more than one game type was associated with levels of problem gambling; after adjustment for demographic variables sport betting and slots increased risks of problem gambling in both samples—one-game and multi-game players; poker and other card games increased risks only for multi-game players; lotteries were protective in both samples, scratch cards only for multi-game players
#10 Yeung and Wraith (2017)	Secondary data analysis of an Australian population survey from 2008; at least 18 years old	11,177 last year gamblers	A series of multinomial logistic and negative binomial regressions	Participation in 11 game types (ordinal level) adjusted for (1) covariates (gender, age, education, residency, language, marital status, employment status), (2) the total number of games played in the past year, (3) total frequency of participation in other gambling activities, (4) interaction effects of age and number of game types or total frequency	Negative binomial regression and logistic regression showed similar results; no interaction effect occured between age and both measures of gambling involvement; after adjustment of covariates and total number of game types or total gambling frequency, large significant risks remained for electronic gambling machines and casino table games
#11 Baggio et al. (2017)	Sample of French adolescents	9910	A series of increasingly complex Quasi-Poisson regressions	Participation in Internet gambling (dichotomous) was adjusted for (1) demographic covariates (age, gender, and parental occupational status), (2) number of game types, (3) sum of gambling days, and (4) both measures	Online gambling remained a significant risk factor after adjusting for covariates, number of game types, and number of gambling days; after including all variables simultaneously the association collapsed and only measures of breadth and depth remained significant predictors
#12 Nelson et al. (2018)	Online survey among residents of Massachusetts; at least 18 years old	274 last year gamblers	A series of bivariate and simultaneous logistic regressions for each game type; finally a hierarchical regression	Participation in 19 game types (dichotomous level) was successively adjusted for (1) patterns of game play (4 factor scores, previously extracted from a principal component analysis of gambling frequency measures), (2) overall depth of involvement (maximum frequency, daily hours, amount gambled, amount lost), and (3) number of game types	All bivariate associations of game type participation and at least one DSM-IV criterion of problem gambling disappeared after adjusting for different measures of gambling involvement; number of game types had the strongest impact on effect reductions; component measures of gambling patterns and overall involvement were strongly interrelated
#13 Castrén et al. (2018)	Secondary data analysis of a Finnish population survey from 2015; at least 18 years old	3555	Stepwise negative binomial regressions for game types after controlling for demographics and overall involvement (number of game types was dropped due to collinearity) from a final simultaneous model	Participation in gambling mode (online; offline) and 12 game types (ordinal level) adjusted for (1) covariates (gender, age), (2) overall expenditure by net income, and (3) overall gambling frequency	After adjusting for demographics and the risk increasing impacts of online gambling, overall frequency and spent percent of income, ascending levels of gambling frequency in scratch games, betting games, slot machines, non-poker games on Finland’s Slot Machine Association online casino, and non-monopoly gambling (mainly online and strongly associated with the number of game types) remained significant risk factors for more gambling related harms
#14 Cavalera et al. (2018)	Online survey among Italian adults; 18–94 years old	4773	Stepwise and simultaneous multinomial logistic regressions	Participating in online games, strategic, non-strategic, and a combination of both were included with (1) demographic covariates (gender, age, marital status, education, parental gambling problems) and (2) number of game types	After simultaneously adjusting for significant predictors of the stepwise regression, number of game types, strategic games (poker, betting), and the combined use of strategic and non strategic games (lottery, slot machines, pull-tabs, bingo), remained significant risk factors for at risk gambling and problem gambling

The studies were selected from a coarse query with “Google Scholar” in September 2018 with the terms [“gambling” and “intensity”], [“gambling” and “involvement”], [“gambling” and “breadth”], or [“gambling” and “depth”]. Once a study was extracted as a candidate, additional checks were conducted in their references and other studies citing them. However, only studies applying linear regression models with ascending levels of complexity were included. The aim of this simple approach was not to be exhaustive, but rather to highlight the state of the art of the linear regression-based modeling approach to evaluate bivariate associations of problem gambling and participating in particular game types.

### Regression-Based Explanations for Associations of Problem Gambling and Game Types

In all selected publications, general or generalized linear regression models (e.g., negative binomial, logistic or multinomial regressions) were applied. However, all of them were based on vastly different samples, ranging from the general population of Australia to patrons of two Las Vegas resort casinos (Table 1). In addition, different outcome criteria have been applied to determine problem gambling. For instance, LaPlante et al. (2011) applied a 3+ symptom cut-off score based on a last year DSM-IV problem gambling assessment, LaPlante et al. (2014) used the Brief Biosocial Gambling Screen, LaPlante et al. (2013) referred to a single item of self-perceived gambling-related problems, and Yeung and Wraith (2017) even compared the impacts of two problem gambling outcomes with different scale levels on parameter estimates. These methodological differences substantially impede comparability of the results.

However, in spite of the methodological variations, the core idea of all data analyses was to examine the statistical association of participation in a specific game type and problem gambling. Therefore, participation in individual game types was applied as a predictor of problem gambling outcomes. This was done either in simultaneous model estimation with all game types and other covariates (e.g., Welte et al. 2009) or in a series of logistic regressions (e.g., Brosowski et al. 2012). In some cases, stepwise or hierarchical procedures were applied to preselect relevant predictors.

The pivotal assumption of this statistical approach is: If a significant positive bivariate statistical association of problem gambling and participating in a specific game type becomes non-significant after adjusting for one or more confounding variables, the former association was spurious and probably caused by a biased estimation. For example, a game type may be more attractive for young and less educated males who are generally more prone to problem gambling (Brosowski et al. 2015; Olason et al. 2015). These more complex linear regression models, which comprise of age, gender, or other demographics as predictors, adjust for such bias.

### Problem Gambling, Game Types, Gambling Behavior and Beyond

In their 2011 paper, LaPlante et al. replicated an analytical approach of Welte et al. (2009) in a British population survey and confirmed that incorporating the *total number of game types* a person played during the last year as a predictor strongly decreased bivariate positive associations of game type participation and problem gambling in applied regression models. Both studies confirmed that considering the full range of gambling activities in addition to participation in one type of particular interest was crucial in explaining problem gambling with regression models. LaPlante et al. (2011) reasoned that “[…] some games might be indicators of unhealthy involvement, rather than critical factors for gambling-related problems themselves” (p. 535). Moreover, ascending levels of gambling involvement across more than one game type could also be indicative of attempts to win back losses made in another (Blaszczynski 2013)—resulting in some kind of “cross-game-chasing-behavior” (chasing constitutes a central symptom in diagnosing disordered gambling; see Shaffer and Martin 2011).

A plausible hypothesis about the explanatory importance of the number of game types involved in as an indicator of overall gambling involvement could be the concept of an “omnivore”, a sociological concept to explain the consumption of numerous cultural activities amongst others with advantaged social positions (Chan 2019; Holbrook et al. 2002). Applied to the field of problem gambling, the specific characteristics of certain game types may become successively less important for individuals with higher levels of problem gambling. Instead of the *specific* content of a game, the thrill of participating in *any* gambling activity becomes more interesting for these gamblers.

Subsequent analyses confirmed the pivotal role of the total number of game types played (i.e., *breadth of involvement*). They also underlined the importance of another construct of overall gambling intensity, the *depth of involvement*. Exemplary measures of depth of involvement include frequency of gambling across all game types during the last year combined (LaPlante et al. 2013) or the number of days a person placed a bet (LaPlante et al. 2014).

### Conflicting Empirical Evidence in Regression-Based Approaches

Regression-based research on problem gambling, game type participation, and measures of gambling consumption has been progressing continuously during the past years. Recent research also examined interaction effects of measures of gambling intensity with age or gender (Afifi et al. 2014; Yeung and Wraith 2017). The pivotal role of measures of overall gambling intensity was largely confirmed. But even in very complex linear regression models, which simultaneously incorporated socio-demographic covariates, *breadth of involvement*, and *depth of involvement* (e.g., Afifi et al. 2014; Haß et al. 2012), certain game types remained significantly associated with problem gambling (Table 1). In particular, participation in casino games, sport betting, or EGMs remained risk increasing predictors in several analyses.

However, in some studies, other game types even became protective factors against problem gambling (e.g., private betting, betting on horses, poker, lotteries, or scratch cards). This further complicates the complex relationship between problem gambling, game types, context, individual predisposition, and behavior (Blaszczynski 2013; Currie and Casey 2007; Shaffer and Martin 2011). Possible reasons for the partially contradictory results about impacts of game types on problem gambling may be found in methodological differences, such as variable operationalization, sample size, measurement, or statistical modeling, but also a substantial matter of different populations, contexts, or jurisdictions.

In sum, the partially inconsistent empirical results of previous sophisticated regression-based studies document the complex relationship between gambling behavior and problem gambling as well as the challenges statistical modeling approaches of this relationship face. Hence, it is a major duty for further research (1) to disentangle the complexity of gambling problems and behavior as precisely as possible and (2) to provide a valid groundwork for evidence-based harm reduction measures referring to particular game types and overall gambling behavior in a given population. In a more practical sense, the inconsistent previous results also show that an unbiased evaluation of the impact of participating in particular game types on problem gambling behavior has to consider (1) demographic characteristics of the gambler and (2) his overall gambling behavior *within and beyond* the examined types of interest (i.e., *depth* and *breadth* of involvement).

### Methodological Challenges in Common Regression-Based Approaches

Including *depth* and *breadth* of involvement in regression-based models, however, not only results in the aforementioned empirical inconsistencies, but also in several methodological problems: First, the additionally inclusion of measures of overall gambling intensity (*breadth* or *depth* of involvement) in a bivariate model that predicts problem gambling by gambling participation in a specific game type only allows for *indirect* appraisals of the importance of the intensity measure. The extent of the decrease in statistical association of game type and outcome may be indicative for such an indirect appraisal (e.g., see changes in risk estimates in Yeung and Wraith 2017). Nevertheless, scalable effect sizes and confidence intervals for such indirect effects are missing.

Furthermore, different measures of consumption behavior are often highly correlated (Currie and Casey 2007; Room 2000). Statistical power of regression models is often strongly affected by such multicollinearity (Tabachnick and Fidell 2007) and parameter estimates may be destabilized. Some empirical examples of high multicollinearity among measures of gambling involvement may be found in the studies from Castrén et al. (2017), Salonen et al. (2018), or Nelson et al. (2018). Castrén et al. (2018) even had to drop the number of game types from their multivariate model to predict the number of gambling harms due to high multicollinearity with other predictors of overall or type specific gambling involvement.

Moreover, including *breadth* and d*epth* of gambling involvement as simple predictors of problem gambling is conceptually questionable, because dimensions of consumption intensity like money or time are not only variables to control for—they are part of the *etiological mechanisms* that may *cause* problem gambling or gambling-related harms (Blaszczynski 2013; Delfabbro and King 2019).

### Parallel Multiple Mediation Models May Overcome Current Challenges

Against this background of empirical inconsistencies and methodological challenges, mediation analyses may solve some of the outlined problems. The core idea of mediation analyses (Hayes 2013, 2018; MacKinnon et al. 2000; Preacher and Hayes 2008) is to explicitly model possible pathways or mechanisms **M** (mediators) by which a predictor variable **X** (last year participation in game type x: yes; no) affects an outcome variable **Y** (problem gambling). In other words, mediation analyses answer the question *how a predictor impacts an outcome* and provide explicit point estimates and confidence intervals for specific mediating mechanisms of problem increasing gambling behaviors. Particularly this aspect qualifies parallel multiple mediation analysis to compare the importance of different plausible mechanisms within and beyond a game type, which may impact problem gambling.

It is worth noting that the depicted previous linear-regression models in their current way of application are similar to an old-fashioned approach of mediation analyses, the so called “causal steps approach” (Hayes 2018; Preacher and Hayes 2008). The criticism on (1) reduced statistical power as well as (2) lacking point estimates and confidence intervals of specific mechanisms that this old mediation approach faced is also warranted in the outlined regression-based studies on gambling behavior. Modern ways of mediation modeling overcome these issues (Hayes 2018). Estimating models with multiple parallel mediators provides explicit information about the most important mediating pathways by which a predictor affects an outcome of interest (Hayes 2018; Preacher and Hayes 2008).

Moreover, percentile bootstrap estimation of indirect effects and their confidence intervals is able to cope adequately with some bias caused by strongly correlated mediators like in the case of measures of gambling intensity. Neglecting correlated mediators can affect the standard errors of the association of mediator and the outcome of interest (so called b-paths, explained in the following section). This in turn affects statistical inference with normal-theory-tests for indirect effects (Preacher and Hayes 2008). Percentile bootstrap confidence intervals to evaluate statistical inference (Hayes 2018) have become widely recommended due to a favorable trade-off between errors of type I (erroneously revealing false indirect effects) and type II (erroneously ignoring true indirect effects).

### The Gambling Consumption Mediation Model

The aim of this study is to provide a new framework of *parallel multiple mediation analyses* that may overcome the methodological challenges of current regression-based models [(1) no explicit statistical information about the importance and confidence of specific mechanisms probably impacting problem gambling, (2) multicollinearity of gambling behaviors, (3) lack of conceptual clarity]. The proposed *gambling consumption mediation model* (GCMM) in the following analyses provides a novel analytical framework that includes (1) last year participation in particular game types as dichotomous predictor variable (yes; no), (2) different proxy measures of gambling involvement (within and beyond the type of interest) as parallel mediators, and (3) a formative index of socio-demographic covariates that commonly increase the risk of problem gambling as another mediator that may pose an additional way via which particular game types impact an (4) outcome of problem gambling. The presented analytical approach of parallel multiple mediator models for each individual game type in the GCMM generates precise information *by which specific indirect effect* participation in a particular game type increases gambling problems. These empirically confirmed mechanisms can be addressed precisely by evidence-based harm reduction measures. Moreover, the applied mechanisms of the model (demographics or gambling behavior) are (1) founded in current research (Table 1) and (2) available in most existing datasets developed to monitor gambling behavior and problem gambling in given populations.

## Method

### Data Set

The data base (N = 7221) of the following analyses was merged from three similar cross sectional representative population surveys from Iceland of the years 2007 (n = 2631), 2011 (n = 1887) and 2017 (n = 2703). Further information about the surveys’ identical methodology can be found elsewhere (Olason et al. 2015). Only non weighted data were employed in the following analyses.

### Variables

The following subchapters will provide an in-depth explanation of the independent variables and mediators (predictors = X; mediators = M) as well as the dependent variable (outcome = Y) and, where necessary, how they were created.

#### Gambling Behavior Within Particular Game Types (Depth Within)

The applied dataset contained measures of gambling behavior with regard to 15 different game types: (1) lotto offline, (2) lotto online on an Icelandic website, (3) EGM offline, (4) scratch cards offline, (5) betting on sport pools offline, (6) betting on sport pools online on an Icelandic website, (7) sport betting offline (fixed odds), (8) sport betting online on an Icelandic website (fixed odds), (9) live betting online on an Icelandic or foreign website, (10) sport betting online on a foreign website, (11) poker offline, (12) poker online on a foreign website, (13) betting on skill games offline, (14) bingo offline, (15) other online gambling on foreign websites (not poker or betting), e.g., EGM, scratch cards, or casino games.

For each of the 15 game types two different variables quantified gambling involvement: (1) *usual spending within*: “How much money did you usually spend each time you gambled on …” (answered in an open-ended format of Icelandic Krona) and (2) *frequency within*: “How often do you play …” (answer in ascending categories: 0 = “never”; 1 = “a few times the past 12 months [once to 11 times]”; 2 = “once to three times a month”; 3 = “once to twice a week”; 4 = “three to six times a week”; 5 = “daily”). All following analyses were only applied to individuals who participated at least in one game type during the last year (n = 4422; 61.3% of the entire sample). Because the open-ended variables of usual spending behavior were prone to biases by outliers, the spending variables were winsorized within the 1st and 99th percentile, which means that values beyond the percentiles were replaced by these values. Frequency variables were not winsorized due to the lower biasing potential of the restricted number of categories. Among the final sample of last year gamblers, missing values in both depth variables were replaced with values of 0 Krona spent or a frequency of 0 (never).

The dichotomous information of last year participation in a particular game type (yes, no) was extracted from the frequency variables and was applied as independent variable (predictor = X) in the following mediation models.

#### Gambling Behavior Beyond Particular Game Types (Depth Beyond)

For each of the 15 game types *gambling frequency beyond* the individually analyzed game type of interest was aggregated in a new variable (gambling frequency beyond type x) by the maximum function across gambling frequency in all 14 other game types except the type of interest. Moreover, for each of the 15 game types *usual spending beyond* the game type of interest was aggregated in a new variable by the maximum function across usual spending in all 14 other game types except the type of interest. The maximum function was chosen to avoid high correlations with the breadth variable, which occur across all game types when using a sum function instead.

#### Multiple Gambling Involvement (Breadth)

For each individual the number of game types that were used at least once during the last year (*breadth*) was summed up to a score ranging from 1 to 15 game types. Individuals with more than 7 game types (99th percentile) were replaced by the value of 7 game types to avoid strong biases by statistical outliers.

#### Propensity of Problem Gambling (Demographic Index)

Among the last year gamblers, a binary logistic regression was used to predict the outcome of being at least at moderate risk of problem gambling (n = 55; 1.2% of the sample) by four demographic predictors: (1) male gender (OR 5.026; *p* ≤ 0.001), (2) three ascending levels of education (OR 0.527; *p* = 0.002), (3) being a single or divorced or widowed vs. being married or living with a spouse (OR 1.671; *p* = 0.068), (4) age in years on a metric level from 18 to 70 years (OR 0.951; *p* ≤ 0.001). The metric propensity score was saved in the dataset. Due to case-wise exclusion of missing values in the binary regression, 130 of 4422 (2.94%) individuals showed missing values in the propensity score. These missing values were replaced by the mean of all other cases. Next to the five mentioned measures of gambling behavior this metric formative index of demographic problem gambling vulnerability served as another mediator. Including this composed index of demographic problem propensity as mediator allows for direct comparison with other mediators like gambling behavior.

#### Problem Gambling (Outcome)

Problem gambling was measured by the nine items (each item with 0–3 point scale) of the Problem Gambling Severity Index (PGSI; Ferris and Wynne 2001) which were aggregated by the sum function (non-gamblers had the value 0). Four groups of gambling behavior were extracted by an updated rule of Currie, Hodgins, and Casey (2013; *p*. 323): (1) “no problem” = 0 points; (2) “low-risk gambling” = 1–4 points; (3) “moderate-risk gambling” = 5–7 points; (4) “problem gambling” = 8 or more points. Among the 4422 last year gamblers, the fractions of the four groups did not differ substantially across the three surveys (*X*^2^ = 12.209; *df* = 6; *p* = 0.057; Cramer’s V = 0.037). In total, the fractions were the following: no problem = 89.9% (n = 3974), low-risk gambling = 8.9% (n = 393), moderate-risk gambling = 0.7% (n = 29), and problem gambling = 0.6% (n = 26). For the parallel multiple mediation analyses individuals with a PGSI sum score larger than 6 (99th percentile) were winsorized by the value 6 to avoid strong biases by statistical outliers.

### Data Analyses

The following analyses were conducted among the sample of N = 4422 last year gamblers only (49.80% male; age = 18–70 years old; mean = 42.57; standard deviation = 14.39; 24.40% primary education, 42.40% secondary education, 33.30% university; 63.20% were married or living with a spouse, 36.80% were single, divorced or widowed).

In a first step of *cohort analyses*, the last year participation (yes; no) in each of the 15 game types across the three years of the different surveys (2007, 2011, 2017) was analyzed by asymptotic Chi Square tests and effect size measures of Cramer’s V (for small cell counts Fisher’s exact test statistic was applied). Moreover, the ordinal or metric mediators (frequency within, frequency beyond, usual spending within, usual spending beyond; multiple gambling involvement and demographic problem gambling propensity) were analyzed across the years by Kruskal–Wallis tests and effect size measures of Eta-Square. The aim of these cohort analyses was to decide whether it was justified to treat the merged dataset as a homogeneous population in the second step of parallel mediation modeling. Not missing relevant cohort differences (error of type II) was more important in this testing situation than detecting a spurious effect (error of type I). Therefore, individual *p*-levels were not adjusted for multiple comparisons.

In a second step, for each of the 15 game types, a *parallel multiple mediation model* was conducted to assess the impact of last year participation in the game type of interest (yes; no) on the winsorized PGSI sum score. The involved mediators of the models were:

- (M1) demographic problem gambling propensity (propensity),
- (M2) number of used game types (breadth),
- (M3) gambling frequency within the type of interest (frequency within),
- (M4) maximum gambling frequency beyond the type of interest (frequency beyond),
- (M5) usual spending within the type of interest (spending within),
- (M6) maximum usual spending beyond the type of interest (spending beyond).

**Fig. 1:**
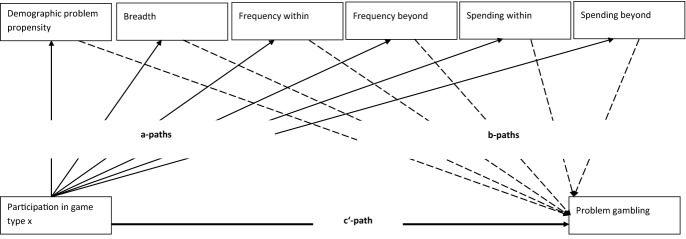
Conceptual map of applied multiple mediator models

A conceptual map of the applied parallel mediation models for each game type is illustrated in Fig. 1. The a-paths estimate the association of last year participation in a game type and the proposed mediating variables. The b-paths estimate the association of the mediator with the outcome score of gambling problems, while simultaneously controlling for other variables (participation and other mediators) in the model. The product of a*b quantifies the *indirect effect* of participating in type X on gambling problems via a specific mediator, e.g., propensity or breadth of gambling involvement. This quantification of particular indirect effects and their confidence intervals gives a precise answer to the question via which gambling behavior (*mediator*) participation in a game type of interest impacts gambling problems. The c′-path estimates the *direct effect* of participating in type X on gambling problems when simultaneously considering all indirect effects of the mediators in the model. Furthermore, the total effect is the sum of all direct and indirect effects on the outcome. All data analyses were conducted with IBM SPSS Statistics Version 25. The multiple mediation models were conducted with PROCESS v3.3 (Copyright [c] 2012–2019 by Andrew F. Hayes), a syntax oriented supplementary tool for advanced mediation analyses in SPSS and SAS, based on ordinary least squares path analysis (documented in Hayes 2018). The number of bootstrap samples was set to 5000, seed was set random, chosen mediation model was number 4. Due to the exploratory nature of this study and the first time application of the GCMM the *p*-levels of individual test statistics within the models were not adjusted for multiple comparisons. Statistical relevance of mediating effects was judged by their 95%-confidence interval (intervals of relevant effects did not include the value 0).

## Results

### Cohort Analyses

Table 2 displays the 12-month participation among last year gamblers (n = 4422) in the 15 game types across the three surveys. The most prevalent game types across all surveys were lotto offline (73.7%), scratch cards offline (31.4%), bingo offline (15.6%), lotto online on an Icelandic website (13.5%), and EGMs offline (12.5%). Due to the large sample size, on a *p*-level of 5% most game types showed statistically significant changes across the years of the surveys. Only scratch cards offline and sport betting offline showed no significant changes. However, the majority of changes were based only on minuscule effect sizes with Cramer’s V values below 0.1.

**Table 2:** 12-month participation among last year gamblers (N = 4422) in 15 game types across three surveys

#	Game type	Statistic	2007 (n = 1398)	2011 (n = 1264)	2017 (n = 1760)	Total	*X*^2^ (*df* = 2); *p*; Cramer’s V
1	Lotto offline	Count	1057	1003	1197	3257	**52.772;** ***p* ≤ 0.001; 0.109**
		% within year	75.6%	79.4%	68.0%	73.7%	
2	Lotto online on an Icelandic website	Count	121	133	344	598	**92.645;** ***p* ≤ 0.001; 0.145**
		% within year	8.7%	10.5%	19.5%	13.5%	
3	Electronic gaming machine offline	Count	210	163	181	554	16.174; *p* ≤ 0.001; 0.06
		% within year	15.0%	12.9%	10.3%	12.5%	
4	Scratch cards offline	Count	428	383	576	1387	2.547; *p* = 0.280; 0.024
		% within year	30.6%	30.3%	32.7%	31.4%	
5	Betting on sport pools offline	Count	107	98	101	306	6.344; *p* = 0.042; 0.038
		% within year	7.7%	7.8%	5.7%	6.9%	
6	Betting on sport pools online on an Icelandic website	Count	30	47	92	169	20.177; *p* ≤ 0.001; 0.068
		% within year	2.1%	3.7%	5.2%	3.8%	
7	Sport betting offline	Count	49	53	49	151	4.478; *p* = 0.107; 0.032
		% within year	3.5%	4.2%	2.8%	3.4%	
8	Sport betting online on an Icelandic website	Count	21	37	66	124	14.543; *p* ≤ 0.001; 0.057
		% within year	1.5%	2.9%	3.8%	2.8%	
9	Live betting online on an Icelandic or foreign website	Count	11	10	81	102	**68.372;** ***p* ≤ 0.001; 0.124**
		% within year	0.8%	0.8%	4.6%	2.3%	
10	Sport betting online on a foreign website	Count	3	15	94	112	**95.93;** ***p* ≤ 0.001; 0.147**
		% within year	0.2%	1.2%	5.3%	2.5%	
11	Poker offline	Count	104	196	169	469	**48.671;** ***p* ≤ 0.001; 0.105**
		% within year	7.4%	15.5%	9.6%	10.6%	
12	Poker online on a foreign website	Count	4	41	36	81	33.033; *p* ≤ 0.001; 0.086
		% within year	0.3%	3.2%	2.0%	1.8%	
13	Betting on skill games offline	Count	44	40	27	111	11.383; *p* = 0.003; 0.051
		% within year	3.1%	3.2%	1.5%	2.5%	
14	Bingo offline	Count	125	192	371	688	**87.559;** ***p* ≤ 0.001; 0.141**
		% within year	8.9%	15.2%	21.1%	15.6%	
15	Other online gambling on foreign websites (not poker or betting), e.g. EGM, scratch cards or casino games	Count	7	17	38	62	15.542; *p* ≤ 0.001; 0.059
		% within year	0.5%	1.3%	2.2%	1.4%	

Cramer’s V: 0.1 = small effect; 0.3 = medium effect; 0.5 = large effect. At least small effects are bold

A small effect emerged for lotto offline with increasing participation from 2007 (75.6%) to 2011 (79.4%) and decreasing participation in 2017 (68.0%). A small effect also emerged for lotto online on an Icelandic website with an increasing participation from 8.7% in 2007 to 10.5% in 2011 and 19.5% in 2017. Moreover, participation in live betting online on Icelandic or foreign websites increased from 0.8% in 2007 to 4.6% in 2017. Also sport betting online on a foreign website increased from 0.2% in 2007 to 1.2% in 2011 and 5.3% in 2017. Poker offline increased from 7.4% in 2007 to 15.5% in 2011 and decreased again to 9.6% in 2017. Participation in bingo offline changed from 8.9% in 2007 to 15.2% in 2011 and 21.1% in 2017. Dividing participation rates of 2017 by rates of 2007 showed that the mean ratio for all 8 offline game types was 1.04 with largest growth values for bingo offline (2.36) and poker offline (1.29). In contrast, the ratio of 2017/2007 prevalence rates for the 7 online game types showed a mean growth rate of 7.06 with largest values for sport betting on foreign websites (24.89), poker online on a foreign website (7.15), live betting on an Icelandic or foreign website (5.85), and other online gambling on foreign websites (4.31).

Kruskal–Wallis tests of the metric and ordinal variables across the years of the surveys also showed significant (*p* ≤ 0.05) but minuscule effect sizes (Eta Square < 0.01) for the majority of inspected attributes (e.g., PGSI sum score or demographic propensity score). The only at least small effects (0.03 ≥ squared eta ≥ 0.01) emerged for the following attributes: The number of game types involved in increased from a mean of 1.660 in 2007 to 1.921 in 2011 and 1.944 in 2017. Frequency within bingo offline, lotto online on an Icelandic website, sport betting online on foreign websites, and live betting online on Icelandic or foreign websites increased from 2007 to 2017. Usual spending within bingo, lotto online on Icelandic websites, sport betting online on foreign websites, live betting online on Icelandic or foreign websites, and poker offline increased from 2007 to 2017. Usual spending beyond a given game type increased for every game type from 2007 to 2017.

In sum, there was an ascending trend in gambling participation, particularly online game types, and slight increases in number of used game types as well as gambling intensity (frequency and usual spending) within and beyond in many or even all game types. However, all relevant trends showed only small effect sizes between 2007 and 2017. Consequently, treating the entire dataset as one homogeneous population in the following analyses represents a justified step.

### Parallel Multiple Mediation Models

Model estimates for unstandardized effects and 95%-percentile bootstrap confidence intervals of the parallel multiple mediation models are presented in Tables 3 and 4.

**Table 3:**
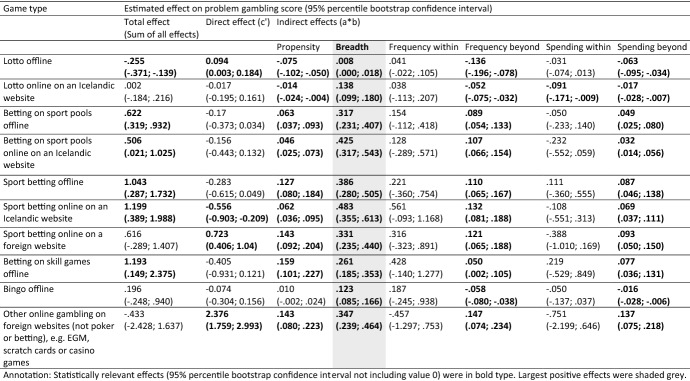
Total, direct and indirect/mediated effects (unstandardized) of participation in 15 game types on problem gambling score via 6 mediators—only game types mostly impacting problem gambling via breadth

**Table 4:**
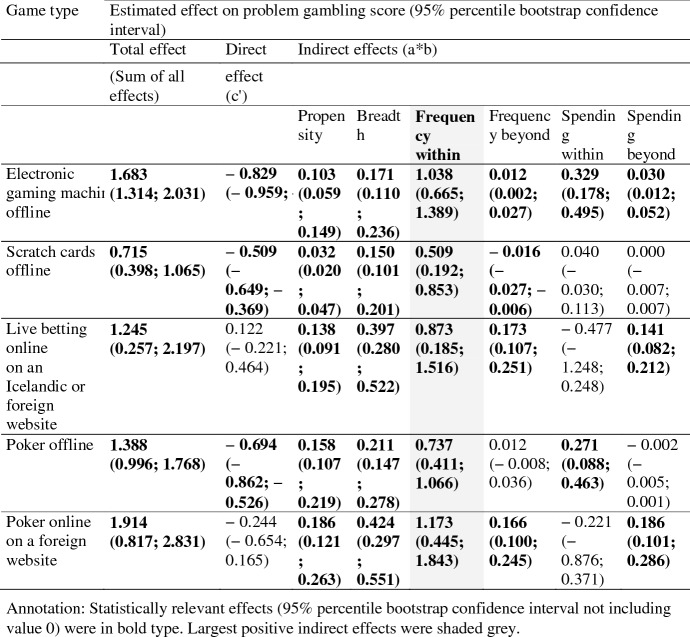
Total, direct and indirect/mediated effects (unstandardized) of participation in 15 game types on problem gambling score via 6 mediators—only game types mostly impacting problem gambling via frequency within

#### Total and Direct Effects

The *total effects* on problem gambling (sum of all direct and indirect effects) of last year participation in 11 of the 15 game types were of statistical relevance (95% percentile bootstrap confidence interval not including value 0) and 10 types were of *problem increasing impact* (positive total effect). Lotto offline was the only game type with a statistically relevant *problem decreasing impact* (negative total effect). For several game types the total impact on problem gambling was not statistically relevant: Lotto online on an Icelandic website, sport betting online on a foreign website, bingo offline, and other online gambling on foreign websites (not poker or betting).

*Direct effects* of game type participation on gambling problems (c′-path: effect of last year participation left when simultaneously considering all indirect effects) became non significant for 8 of the 15 game types. Statistically relevant problem increasing direct effects of last year participation when simultaneously considering all other mediating impacts were left only for lotto offline, sport betting online on a foreign website, and other online gambling on foreign websites (not poker or betting). Problem decreasing direct effects occurred for sport betting online on an Icelandic website, EGMs offline, scratch cards offline, and poker offline.

#### Indirect Effects

However, total and direct effects are only of minor interest in the parallel mediation analyses at hand that try to answer the following question: By which mediator (gambling behaviors or demographic propensity) is last year participation in game type X impacting gambling problems most severely? This aspect can explicitly be tackled by the quantification of *specific indirect effects* (a*b-paths) which provides an effect size and confidence intervals for each mediator, while considering the direct path and all other mediators (indirect paths) within the model simultaneously. The metric of the unstandardized effects is determined by the scales of the X (last year participation 1 = yes; 0 = no) and Y variables (winsorized sum score of gambling problems), which both were similar across all analyzed game types. Hence, the size of the indirect effects is comparable across the mediators.

A close look at the specific indirect effects revealed two distinct profiles of decisive mediators impacting gambling problems within the different game types: (1) mostly via *breadth* or (2) mostly via *frequency within*.

Table 3 summarizes the information about game types which affected gambling problems mostly (largest, statistically relevant positive mediating effect within the game type of interest) via the mediator *breadth* (number of game types involved in). Breadth was the strongest mediating mechanism of gambling behavior on gambling problems for participating in:

1. lotto offline,
2. lotto online on an Icelandic website,
3. betting on sport pools offline,
4. betting on sport pools on an Icelandic website,
5. sport betting offline,
6. sport betting online on an Icelandic website,
7. sport betting online on a foreign website,
8. betting on skill games offline,
9. bingo offline and
10. other online gambling on foreign websites (not poker or betting).

Table 4 gives an overview over the game types which affected gambling problems mostly (largest, statistically relevant positive mediating effect) via the mediator *frequency within*. Frequency within was the strongest mediating mechanism of gambling behavior on gambling problems for participating in:

1. EGMs offline,
2. scratch cards offline,
3. live betting online on an Icelandic or foreign website,
4. poker offline and
5. poker online on a foreign website.

The decision about the most important mediating gambling behavior was unequivocal in almost all game types. For several game types there was only one relevant positive indirect effect like breadth for lotto offline or lotto online on an Icelandic website (positive a*b-path). All other mediating mechanisms did not show statistically relevant effects for these two game types or even showed problem decreasing effects such as propensity for lotto offline or online on an Icelandic website (negative a*b-path).

But these negative effect sizes did not mean that the mediating path propensity was not associated with gambling problems for individuals that participated in lotto (positive b-path). Rather, the negative effect was the consequence of a negative association of gambling lotto offline and demographic problem gambling propensity (negative a-path). In other words: Individuals who participated in lotto showed a reduced level of demographic propensity for gambling problems (because lotto gamblers were probably more likely female, older, better educated etc., and thus the demographic propensity of being a problem gambler was lower). Consequently, the product of a*b for the mediator propensity among individuals participating in lotto offline showed negative effect sizes. Hence, this mediator could not be an important problem increasing mechanism for lotto offline, even if it was positively associated with gambling problems in general.

This fact also holds true for the mediators frequency beyond or spending beyond among lotto or any other negative indirect effects among other game types. For some configurations of game types and mediators, there remained single negative b-paths on a statistically relevant level (95% confidence intervals of the single b-paths were below the value of 0) in the following situations: (1) betting on sport pools online on an Icelandic website via spending within; (2) live betting online on a foreign or Icelandic website via spending within; (3) sport betting online on foreign websites via spending within; (4) other games online (not betting or poker) on a foreign website via frequency within; (5) other games online via usual spending within. These *problem reducing impacts* were not plausible from a theoretical position (the included mediators consistently followed the implicit assumption: increasing levels of the mediator also increase gambling problems). However, in all five depicted situations with statistically relevant negative single b-paths the products of a*b-paths were not statistically relevant. Hayes (2018, p. 520) emphasized that it is not the single a- or b-path that explicates a relevant mediating mechanism but only the product of a*b. Therefore, single a- and b-paths were not illustrated or discussed in detail.

A simple descriptive rule of thumb to determine the most important mediator among a game type is to divide the largest statistically relevant and positive effect by the second largest relevant and positive effect within a game type (presented ratios are based on calculations of not rounded metrics). In almost all game types, this ratio was between values of 3.96 (betting on sport pools on an Icelandic website) and 2.20 (live betting online on an Icelandic or foreign website), indicating distinct orders of most important mediating mechanisms (effect size of the most important mediator outperforming the second by at least factor 2 in 14 of 15 game types). The only exception from this order was betting on skill games offline, with a ratio of only 1.64 between the mediators breadth and propensity, indicating a more equivocal profile of most important mediators among this game type. Participation in any other analyzed game type was unequivocally most strongly mediated either via (1) *breadth* of involvement (10 game types) or (2) via *frequency of gambling within* that type of interest (5 game types).

However, the remarkable positions of the two mediators breadth and frequency within for some game types did not disqualify the other mediators in the GCMM as redundant. On the contrary: Propensity and frequency beyond both showed statistically relevant specific indirect effects in 14 of 15 game types, spending beyond in 13 of 15 game types, and spending within in 3 of 15 game types.

## Discussion

The presented GCMM provides, for the first time, an analytical frame work to disentangle the complex network of (1) last year participation (yes; no) in particular game types, (2) an outcome of problem gambling and different proxy measures of (3) socio-demographic problem gambling propensity, (4) overall gambling involvement (number of game types used, maximum gambling frequency beyond game type, maximum usual spending beyond game type), and (5) game type-specific gambling involvement (gambling frequency within game type, usual spending within game type). The chosen approach of parallel multiple mediation analyses allowed for explicit quantification of the behavioral or interpersonal mechanisms, by which participation in specific game types impacts the outcome of problem gambling. Cohort analyses of the three merged Icelandic surveys warranted treating the empirical dataset as homogeneous population even though the number of used game types increased slightly and online game types showed continuously increasing levels of participation.

A short summary of 14 peer-reviewed regression-based studies outlined inconclusive results as well as methodological and conceptual challenges that can partially be resolved by the chosen approach of parallel multiple mediation analyses for each particular game type with the proposed mediating mechanisms characterizing gambling behavior and demographic problem gambling propensity. The state-of-the-art percentile bootstrap procedure to estimate effects and confidence intervals of mediators is able to cope adequately with unspecified correlated error terms (multicollinearity of mediators), providing robust estimates of indirect effects with adequate errors of type I and II. Inconsistent results of former research may be partially caused by a lack of such robustness and statistical power of very complex models with many highly correlated predictors. Consequently, statistical *p*-levels within the parallel mediation analyses at hand were not adjusted for multiple testing, in order to uphold statistical power of the first time application of the GCMM. Further research may apply a more confirmative approach of model testing, including model-fit-statistics, but the aim of this study was rather exploratory.

Treating different attributes of problem gambling behavior or propensity as *mediators* appears to be conceptually more convincing than the commonly applied regression-based models with many covariates, because gambling behavior in terms of regularity, diversity, and spent money or time may be part of the etiological process of developing gambling-related harms and problems—not only a covariate one has to adjust for statistically.

Of course, mediation analyses are mathematically also regression-based, but the product-term a*b to quantify indirect effects provides additional information. Mediation analyses enabled us to quantify effects and confidence intervals to make precise evaluations of the most important mediating mechanisms by which behavior particular game types impact gambling problems. This information is of notable interest for testing competing models or theories (Hayes 2013, 2018; Preacher and Hayes 2008) and for evidence based gambling regulation policy and harm prevention.

The outlined analytical potential of mediation analyses was combined with a very elaborate process of feature-engineering for proxy measures of gambling behavior which distinguished mechanisms of demographic propensity, usual spending and frequency *within* and *beyond* as well as the number of game types a person was involved in. The applied maximum function to aggregate the beyond variables was chosen to curtail high correlations with the breadth variable, which occur by the alternative sum function across all game types. Further research is needed to make an informed choice about the most appropriate aggregation, because mean or median functions could also be useful proxies. However, the combination of (1) mediation analyses and (2) precise feature-engineering revealed explicit estimates and insights which were mostly concealed or at least only implicit in former research.

The empirical findings of the GCMM showed that last year participation in (1) EGMs offline, (2) scratch cards offline, (3) live betting online on Icelandic or foreign websites, (4) poker offline, or (5) poker online on foreign websites impacted gambling problems mostly via mechanisms of gambling *frequency within* these types and not by the breadth of involvement or any other mediating variable of the model. Interestingly, results support findings of previous studies in which some of these types still showed explanatory power for gambling problems despite lots of other covariates in the models [(1) for EGMs or comparable game types offline [slot machines, virtual gaming machines or video lottery terminals] see: Afifi et al. 2010, 2014; Castrén et al. 2018; Cavalera et al. 2018; Haß et al. 2012; LaPlante et al. 2011; Scalese et al. 2016; Yeung and Wraith 2017; (2) for scratch cards or comparable game types offline [instant win tickets] see: Afifi et al. 2010; Castrén et al. 2018; (3) for live betting see: Brosowski et al. 2012; LaPlante et al. 2014; (4) for poker or card games see: Brosowski et al. 2012; Cavalera et al. 2018; Welte et al. 2009]. It is worth noting that several studies revealed an adjusted risk increasing effect of offline casino games that are not slots (Afifi et al. 2010, 2014; Haß et al. 2012; Yeung and Wraith 2017). This category may cover the impacts of poker or card games (conflated with other table games) but more precise information was not available. This fact illustrates the importance of distinct feature-engineering for each individual game type to get precise results and to make more adequate decisions in a regulatory context. Therefore, the GCMM was applied even to game types with low participation rates. Further research is needed to protect these results against the flaws of sampling variance or biased parameter estimates, which may emerge from this more precise and exhaustive approach (no combination of any game types, evaluation of all game types with available data).

To our knowledge, the GCMM represents the first analytical approach that provides explicit estimates of indirect effects and confidence intervals of the most important mediating mechanisms of a game type of interest impacting problem gambling. This data-driven information facilitates a valid evaluation of risk increasing mechanisms of game types and complements former evaluation tools (Meyer et al. 2011). Of course, the breadth of involvement remained a pivotal mechanism impacting gambling problems in all analyzed game types. This is in line with former research (see Table 1) and is also plausible due to the introductorily proposed concepts of gambling omnivores or cross-game-chasing-behavior.

Nevertheless, 5 of the 15 game types at hand (EGMs offline, scratch cards offline, live betting online on Icelandic or foreign websites, poker offline, or poker online on foreign websites) impacted gambling problems mostly in a qualitatively distinct way different from the breadth mechanism—namely via gambling frequency within the type of interest. Furthermore, *frequency within* also outperformed the size of usual spending as a problem increasing mechanism within these game types. This result challenges the importance of money in adequately assessing problem gambling behavior (Currie and Casey 2007). Obviously, regular gambling involvement in these game types was a stronger problem increasing mechanism than the extent of usual spending. Further research is needed to substantiate the results and to preclude methodological artifacts (e.g., possible artifacts associated with the number of categories of an applied mediator). Moreover, a combined attribute of usual spending in relation to disposable income (Currie et al. 2006; Currie and Casey 2007) may serve as a more detailed and valid indicator than usual spending within a session.

It is worth noting that the results are in line with data from Germany (Brosowski et al. 2015), which revealed robust associations between different outcomes of gambling problems and probable low-risk-gambling thresholds for *type*-*specific* measures of gambling frequency within EGMs and poker. Obviously, the GCMM has the potential to conceptually support, model and specify a promising line of research on gambling consumption that tries (1) to explicate the dose–response-relationship of gambling intensity and measures of gambling related harm and (2) to establish evidence based thresholds of low-risk gambling intensity by risk-curve- and receiver-operating-characteristic-curve-analyses (Brosowski et al. 2015; Currie 2018; Currie et al. 2006, 2017).

Additionally, some authors recently noted (Abbott 2017) or provided empirical evidence (Markham et al. 2016) that general thresholds of low-risk gambling across all game types are questionable due to varying type-specific “toxicity” (Abbott 2017, p. 2021), for instance in EGMs. It is theoretically plausible that these toxic game types, due to their inherent contextual or structural characteristics (Meyer et al. 2011), lead to increased gambling frequency within. The GCMM explicitly models these suggestions and empirically answers the question for which gambling products form-specific thresholds may be quite useful. In other words, for a game type which mainly impacts gambling problems via breadth, *within*-*measures* of harm reduction that address gambling frequency within the type may not be the most effective and efficient approach. On the other hand, for a game type with high toxicity, which mostly impacts gambling problems via mechanisms *within* that type, game type-specific thresholds of low risk gambling may be very useful. The empirical findings of reliable game type specific thresholds for EGMs and poker (Brosowski et al. 2015) foster this rationale. Moreover, other methods of harm prevention which address excessive type-specific involvement like mandatory pre-commitment or tracking-systems (Hancock and Smith 2017; Ladouceur et al. 2012) or operator/venue based early detection by robust rules of thumb (Brosowski et al. 2015) may be effective for toxic game types which impact gambling problems mostly via frequency within.

Consequently, the GCMM seems to reduce large fractions of the complexity of type-specific and unspecific gambling behavior, socio-demographic predispositions and problem gambling on a manageable level and thus provides useful information for gambling regulation or harm prevention. Moreover, the GCMM refutes the complexity argument, which is often made by gambling industry as part of a lobbying-strategy (Petticrew et al. 2017). The explicitly mentioned most important harm mechanisms can be addressed effectively by adequate measures of harm reduction. For game types that affect problem gambling mostly by mechanisms *beyond* the type of interest (by breadth, frequency, spending beyond, or propensity), supply and access reductions may be effective due to the simple and convincing mechanisms of the total consumption model (Meyer et al. 2018; Rossow 2018). Additionally, means to recoup individual self-control like venue based self-exclusion (Gainsbury 2014) or multi-venue or operator self-exclusion (Gainsbury 2015; Pickering et al. 2018) may provide useful measures of harm reduction. Of course, this also holds true for game types mostly impacting via mechanisms within.

Despite the statistical relevance of the six applied mediators for many game types, some *direct effects* (c′-path) of game type participation on gambling problems still showed stabile risk increasing or decreasing impacts. For instance, (1) lotto offline, (2) sport betting online on a foreign website, or (3) other online gambling (not betting or poker) on a foreign website showed *direct problem increasing* impacts. On the contrary, (4) sport betting online on an Icelandic website, (5) EGMs offline, or (6) scratch cards offline or (7) poker offline showed *direct problem decreasing* impacts (some kind of protective effect). Obviously, the applied mediators of the GCMM were not able to disentangle the complexity of gambling behavior exhaustively and other potential mediators may be at work that can additionally explain direct effects thoroughly. A mediation model which includes all relevant mechanisms will dissolve all direct effects (c′-paths) completely. Hence, future research on mediating mechanisms on problem gambling should include additional variables, for instance psychological or psychiatric attributes or other operationalizations of propensity that include comorbidities or attributes of personality. For example, the problem *increasing* direct effect of lotto may be attributable to demographics and health related behavior (Reid et al. 1999) or demographics and psychological characteristics (Burns et al. 1990), which are currently not part of the GCMM. Beyond risk increasing or decreasing characteristics of the gambling *individual*, the gambling c*ontext* may be another important aspect to dissolve the direct effects which were currently left. For instance, the protecting direct impact of participating in sport betting online on an Icelandic website may be attributable to mostly conventional fan-based betting behavior. When simultaneously controlling for all other mechanisms in the GCMM, a kind of enthusiasm for sports or a team may pose a protective effect. Protective effects of EGMs, scratch cards and poker *offline* may be attributable to an interpersonal and social character of these terrestrial game types. On the contrary, problem *increasing* impacts of sport betting or other *online* gambling *on foreign websites* may be attributable to a lack of such fan-based or social elements, or again a consequence of interpersonal attributes or vulnerabilities (Gainsbury 2015). However, further mediation research is needed to substantiate these speculations about personal or contextual effects with statistical estimates of these mechanisms and to dissolve direct effects from the model completely. Subsequently, regulatory actions could be based on results of this expanded GCMM—again simply by addressing the most important mechanisms. Therefore, the direct effects still left in the current model should not be over-interpreted at this early point of research in mediating gambling behavior [due to the dichotomous level of the predictor, the applied effect sizes of direct and indirect effects must not be compared directly (Hayes 2018)].

The focus of the current GCMM was on mechanisms of (1) *gambling behavior* and (2) *demographic propensity*, of which information is available in most monitoring datasets, and via which game type participation may positively impact ascending levels of problem gambling. Therefore, the result section was mainly narrowed to the *problem increasing mechanisms*, mostly disregarding problem decreasing mechanisms. Further research is needed to elaborate potential beneficial mechanisms or protective factors. Further research should also specify the applied descriptive rule of thumb (ratio of largest and second largest positive indirect effect) to discriminate both emerging mediating profiles (mostly via *breadth* or via *frequency within*) by inferential statistics. Moreover, structural equation models could be applied to explicitly include correlated mediators into the statistical model.

The aggregation of demographic information into a mediating propensity score allows for precise comparison of the indirect effect sizes of demographics and behavior. However, in other situations applying demographics as covariates or moderators may be more adequate.

The analyses at hand treated gambling problems as a continuously ascending scale. Treating gambling problems as a nominal attribute with different estimates for qualitatively different groups of low, medium and high risk gamblers may be a fruitful extension of the GCMM. Additionally, modeling outcomes of gambling-related harms (Delfabbro and King 2019) via the GCMM may provide useful insights in gambling behavior and broader aspects of public health.

Interestingly, the ratio of online and offline game types was similar in both groups of qualitatively distinct patterns of most important mediators (mostly via breadth = 5/10 online types; mostly via frequency within = 2/5 online types). Therefore, the proposed way to distinguish game types statistically by their most important risk increasing mechanism was obviously unaffected by cursory classification in online or offline game types. At the same time, particularly for Internet gambling, it is worth noting that (1) the way or *mode of access* and (2) certain *access configurations* (e.g., mixed online and offline participation) could have remarkable impacts on the GCMM, but are currently not considered adequately (Gainsbury et al. 2012, 2015, 2016; Wardle et al. 2011). For instance, different modes of access could moderate some or all suggested mediation paths. In this vein, future research has to prove the proposed GCMM for moderated mediation or via multi-group-analyses across different subgroups to evaluate the applicability in different contexts and samples. Nevertheless, multiple mediation analyses of gambling behavior in large population samples may provide a sound starting point for further conceptual models of problem gambling behavior and for the new questions of *how people gamble instead of what they gamble*, especially in the context of new gambling technologies (Gainsbury 2015; Wardle et al. 2011).

In general, the GCMM is in line with other recent research that sheds light on relevant gambling issues by advanced methods of statistical modeling (e.g. Ifrim 2015; Markham et al. 2016; Philander and MacKay 2014; Rintoul et al. 2013). However, it has to be taken into account that the chosen way of parallel multiple mediation analyses does not represent the only way to reduce complexity in multivariate gambling behaviors. There are approaches of clustering or latent class analyses to handle heterogeneity of gambling populations (Challet-Bouju et al. 2015; Goudriaan et al. 2009; Savage et al. 2014; Studer et al. 2016; Wardle et al. 2011). Despite this progress, the methods of unsupervised learning mentioned above are not directed to the outcome of problem gambling in building up subgroups from the data. Therefore, they do not provide such explicit and precise information about risk increasing mechanisms like the GCMM. A very smart alternative to complex statistical modeling was recently conducted by Binde et al. (2017) who only applied prior hypotheses and very robust statistical methods to similar research questions like the GCMM. Such knowledge triangulation from different modeling approaches will further deepen our understanding of the complexity of gambling behavior.

## Limitations

There are several limitations of the study at hand that have to be considered. First, the survey data may suffer from the well-known issues associated with self-reports like recall bias or information management (Currie and Casey 2007). Thus, further research should apply the GCMM on other datasets, for instance prospective actual behavioral data.

Second, despite the inclusion of many behavioral components and probable confounding impacts in the mediation models, the data at hand are cross sectional and the lack of temporal precedence of behavior before gambling problems does not allow for causal inference. Therefore, future research should apply the GCMM on longitudinal data. However, we share the position of Hayes (2018) that mediation is by definition a mathematical phenomenon of cause and effect—even if applied “only” in cross sectional data. The current results of such data are consistent with theory as well as previous research and thus provide a useful background of future mediation analyses with prospective data.

Third, the parameter estimates of the proposed models are conditioned by the mediators finally included. The applied mechanisms were theoretically plausible and derived from current research. However, further research has to negotiate which mediators and operationalizations are obligatory and which are not, to adequately delineate the complex interaction of game type and the attributes or behavior of an individual. For instance, behavioral markers from tracking data with high temporal resolution (Adami et al. 2013) may substantially complement the current mediators of the GCMM by including facets of binge like gambling behavior (Nower and Blaszczynski 2003).

Fourth, despite attempts to reduce effects of outliers by winsorization, the applied OLS regression may be biased by positively skewed predictors, mediators, and outcome. Hence, results have to be replicated with other ways of parameter estimation.

Fifth, core assumptions of the applied mediation models are linearity and additivity of model terms. Further research is needed to expand these simple models adequately, because more complex functions are conceivable, e.g., non-linear or serial mediation models.

Finally, model application in other jurisdictions and countries will increase the generalizability of the model and its results. It should be noted that the proposed GCMM in its core dimensions is only based on variables that are available in the most datasets created for purposes of monitoring (i.e., gambling problems, behavior, and demographics). Therefore, future secondary data analyses on such datasets with the proposed modeling procedure of this study have the potential to replicate and extend the suggested blueprint at hand in different countries with diverse gambling products, contexts, and regulations.

## Conclusion

This study addressed several limitations in previous research on the complex interactions of participating in different game types, problem gambling, and individual gambling behavior or vulnerabilities. Taken together, the proposed GCMM overcomes current methodological challenges by (1) a parallel multiple mediation approach in combination with (2) sophisticated feature engineering of gambling behavior and provides explicit information about the mechanisms by which participation in specific game types mostly impacts problem gambling—either mostly within or mostly beyond the type of interest.
